# Distribution patterns of arterial affection and the influence of glucocorticoids on ^18^F-fluorodeoxyglucose positron emission tomography/CT in patients with giant cell arteritis

**DOI:** 10.1136/rmdopen-2022-002464

**Published:** 2022-08-11

**Authors:** Leander Malich, Falk Gühne, Tobias Hoffmann, Ansgar Malich, Tobias Weise, Peter Oelzner, Gunter Wolf, Martin Freesmeyer, Alexander Pfeil

**Affiliations:** 1 Department of Internal Medicine III, Jena University Hospital – Friedrich Schiller University Jena, Jena, Germany; 2 Clinic of Nuclear Medicine, Jena University Hospital - Friedrich Schiller University Jena, Jena, Germany; 3 Institute of Diagnostic Radiology, Suedharz-Hospital Nordhausen, Nordhausen, Germany; 4 Biocontrol Jena, Jena, Germany

**Keywords:** giant cell arteritis, glucocorticoids, inflammation

## Abstract

**Background:**

Giant cell arteritis (GCA) with the involvement of extracranial vessels is increasingly coming into focus. Isolated aortic involvement in the acute phase of GCA is probably more frequent than estimated because only a minority of patients show typical symptoms. ^18^F-fluorodeoxyglucose positron emission tomography/CT (PET/CT) is a reliable imaging tool to diagnose patients with extracranial GCA. The aim of this retrospective study was to quantify arterial involvement at the onset of a newly diagnosed GCA by PET/CT and to evaluate the influence of glucocorticoid (GC) treatment on the diagnostic performance of this imaging technique.

**Methods:**

The study included 60 patients with GCA at the onset of a GCA. All patients had undergone a PET/CT scan. 44 patients were GC naïve and 16 patients received GC.

**Results:**

The most affected arteries were the ascending aorta (72%), followed by the brachiocephalic trunk (62%), aortic arch (60%) and descending aorta (60%). The aorta and its branches showed an inflammatory involvement in 83.3% of patients. A singular affection of the aorta and the brachiocephalic trunk was revealed in 20% of cases. GC-naïve patients (95.5%) had more frequently affected arteries compared with GC-treated patients (50%).

**Conclusion:**

Our study showed the frequent involvement of the thoracic aorta and brachiocephalic trunk in patients with GCA using PET/CT. Since these vascular compartments cannot be visualised by ultrasound, we advocate screening imaging of the aorta with PET/CT when GCA is suspected. Because the use of GC is associated with a marked decrease in the inflamed vascular segment in GCA, PET/CT should be performed as soon as possible.

**WHAT IS ALREADY KNOWN ON THIS TOPIC**

- Giant cell arteritis (GCA) manifesting in large arteries, that is, the aorta and proximal branches, is increasingly recognised as an important GCA subtype and can be associated with serious complications.
- Systemic symptoms and an acute inflammatory blood profile may be the only presenting features.

**WHAT THIS STUDY ADDS**

- Because the aorta cannot be reliably assessed with ultrasound, ^18^F-fluorodeoxyglucose positron emission tomography/CT (^18^F-FDG PET/CT) is a useful tool to detect large-vessel involvement showing the ascending aorta, the aortic arch and the descending aorta, as well as the supra-aortic branches being the most affected arteries.
- Cumulative glucocorticoid (GC) dose and not the single GC dose is associated with a fast reduction of inflammation; ^18^F-FDG PET/CT should be performed as soon as possible.

**HOW THIS STUDY MIGHT AFFECT RESEARCH, PRACTICE OR POLICY**

- Imaging of the aorta using ^18^F-FDG PET/CT without GC therapy should be performed if GCA is suspected and when there are hints for extracranial GCA because it allows the detection of large-vessel involvement.

## Background

Giant cell arteritis (GCA) is the most common idiopathic systemic vasculitis in adults over 50 years of age with incidence rates in western countries of around 10 cases per 100 000 people.^1^ The immune-mediated large-vessel vasculitis (LVV) is affecting large arteries, namely the aorta and its branches, especially the superficial temporal artery.^2 3^ Temporal arteritis is the well-known clinical phenotype of this so-called cranial GCA, characterised by acute temporal headache, scalp tenderness, jaw and limb claudication, and visual deficits with the risk of irreversible visual loss.^2^ Colour Doppler sonography (CDS) of the temporal and axillary arteries is recommended as the first imaging modality in patients with suspected predominantly cranial GCA.^4^

However, recent studies have shown that GCA quite often also features inflammation of the aorta and its primary branches with or without involvement of the temporal arteries.^5^ Therefore, patients with involvement of extracranial, supra-aortic arteries (especially axillary artery) represent a clinically significant and distinct subgroup of GCA, referred as extracranial GCA.^2 6^ Depending on the degree of stenosis as a result of the inflammatory involvement of the aorta and upper extremity arteries, the symptoms can range from claudication of the arms to pain at rest and paraesthesia to gangrene of the fingers and hand. However, systemic symptoms (eg, night sweat, low-grade fever, malaise, fatigue) and an acute inflammatory blood profile can be the only manifestations of extracranial GCA. Because of the paucity of specific signs, extracranial GCA can be overlooked, resulting in a *delay* of diagnosis and treatment. High-dose glucocorticoids (GCs) are the cornerstone of GCA therapy and should be initiated as early as possible to rapidly control disease manifestations and prevent complications.

Due to the non-specific clinical presentation, extracranial GCA is usually diagnosed by imaging such as CT angiography, magnetic resonance angiography of the aorta and ^18^F-fluorodeoxyglucose positron emission tomography/CT (^18^F-FDG PET/CT).^7^

In the last 10 years, PET/CT has become increasingly recognised as an important tool for rheumatologists in the assessment of LVV and GCA.^8 9^ Based on the ability to detect enhanced glucose uptake from high glycolytic activity, ^18^F-FDG PET/CT supports localising arterial wall inflammation and enables distinguishing vasculitis from atherosclerotic lesions as well as inflammation of periarticular and extra-articular synovial structures in polymyalgia rheumatica (PMR).^9^ Given these distinct advantages, ^18^F-FDG PET/CT has been recommended as a tool for the detection of mural inflammation and/or luminal changes in extracranial arteries to support the diagnosis of GCA by both the American College of Rheumatology (ACR)^10^ and the European Alliance of Associations for Rheumatology.^4 11^

The aim of our retrospective study was to quantify arterial vessel involvement in patients with new-onset GCA by ^18^F-FDG PET/CT and to evaluate the impact of GC treatment on diagnostic accuracy of ^18^F-FDG PET/CT.

## Methods

### Study population

This retrospective study included patients with recent-onset GCA, diagnosed at the Department of Internal Medicine III at the Jena University Hospital–Friedrich Schiller University Jena/Germany according to the 1990 ACR Classification Criteria for GCA.^12^

A CDS examination of different cranial arteries was performed in all patients who presented to our rheumatology clinic with clinical suspicion of GCA, showing in 20 cases a temporal arteritis (cranial GCA).

In general, clinical symptoms were grouped as follows:

- A: cranial symptoms (headache, jaw claudication, visual impairment, amaurosis fugax).
- B: PMR symptoms (symmetrical pain of both shoulders or hips (shoulder and pelvic girdle)).
- C: systemic symptoms (fever, night sweat, weight loss, fatigue, cough).

The diagnosis of an extracranial GCA was based on the clinical presentation, elevated inflammatory laboratory parameters (C reactive protein (CRP), erythrocyte sedimentation rate (ESR)) and positive PET/CT findings. All patients had undergone an ^18^F-FDG PET/CT scan between 2010 and 2019.

### ^18^F-FDG PET/CT imaging technique and protocol

PET/CT was performed according to internal standard operating procedures. All examinations took place according to clinical indications and independent of study-specific aspects. An activity of approximately 250 MBq ^18^F-FDG was administered intravenously; activity was increased for overweight patients. Furosemide 20 mg was injected additionally to accelerate diuresis. After an uptake time between 60 and 120 min (first 30 min in resting conditions), scans were performed in supine position from skull base to proximal thigh; scan region was enlarged in case of clinical suspicion of distal involvement. All examinations were conducted at the same Biograph mCT 40 (Siemens Healthineers, Erlangen, Germany). A low-dose CT for co-registration and attenuation correction was performed using the sinogram-affirmed reconstruction system CARE Dose 4D (Siemens Healthineers); additional diagnostic contrast-enhanced CT scans were performed if necessary, but were not considered for study evaluation. PET parameters were scanning time of 2 min per bed position and iterative TrueX reconstruction (Siemens Healthineers).

### ^18^F-FDG PET/CT imaging interpretation

All ^18^F-FDG PET/CT images were analysed in a study-specific second reading by one experienced specialist in nuclear medicine, who was blinded to clinical and pathological findings. The diagnosis of GCA was based on the presence of an active inflammation in the vascular wall of different arterial segments by using the maximum intensity projection of ^18^F-FDG PET/CT. The following vascular regions were examined:

#### Central arteries

- Ascending aorta
- Aortic arch
- Descending aorta
- Abdominal aorta
- Brachiocephalic trunk

#### Peripheral arteries

- Subclavian arteries
- Axillary arteries
- Common carotid arteries
- Common iliac arteries
- Femoral arteries

In contrast to peripheral vascular regions, the central arteries are not accessible to peripheral imaging diagnostics such as ultrasound.

Inflammation of the arterial vessel wall was evaluated by comparing the ^18^F-FDG uptake of the arterial vessel wall with the background liver uptake. The maximum ^18^F-FDG uptake of the vessel wall was determined from all vessel sections using maximum standardised uptake values (SUV_max_). This was related to the maximum ^18^F-FDG uptake of the liver, which was calculated by means of three standardised volume of interest (two in the right liver lobe and one in the left liver lobe) and logarithmised to base 2 to allow more accurate discriminatory power in smaller areas. SUV were calculated using the following equation:

$${\text{SUV}}_{\max}\text{=}\log_{\text{2}}\left({\text{SUV}}_{\text{vessel-max}}/{\text{SUV}}_{\text{liver-max}}\right)$$

We used the qualitative visual scoring method comparing the vascular ^18^F-FDG uptake with the liver uptake (vascular to liver uptake ratio) as recommended by Stellingwerff *et al*.^13^ Briefly, a vascular ^18^F-FDG uptake higher than the liver uptake (positive value) was interpreted as inflammation of the vessel wall, whereas a lower vascular ^18^F-FDG uptake was considered ‘negative’ for vasculitis.

### Statistical analysis

**Figure 1:**
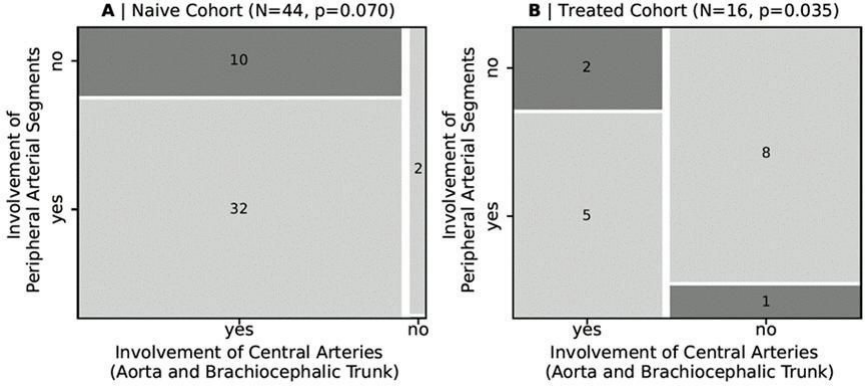
Involvement of arteries, differentiated regarding central segments and peripheral arteries as well as glucocorticoid (GC) treatment ((A) GC-naïve patients and (B) GC-treated patients).

**Figure 2:**
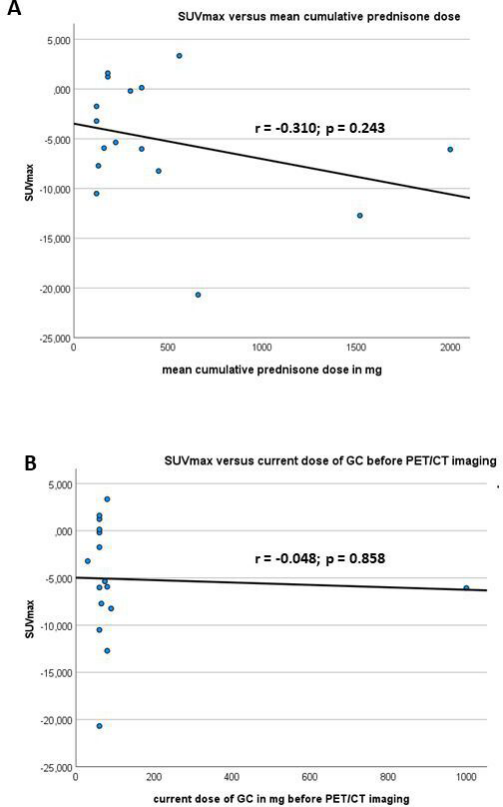
Comparison of and SUV_max_ as well as the (A) cumulative and (B) current dose of GC before PET/CT imaging was performed. GC, glucocorticoid; PET, positron emission tomography; SUV_max_ maximum standardised uptake values.

Descriptive statistics were performed. Computations were carried out by using the programming language python (V.3.6.9) and the additional packages numpy (V.1.16.2), pandas (V.0.25.0) and statsmodels (V.0.11.1). Data visualisation was carried out using the packages matplotlib (V.3.3.0), statsmodels (V.0.11.1) and seaborn (V.0.9.0). P values of <0.05 were considered as statistically significant. For figure 1, a Fisher’s exact test was used. Concerning figure 2, comparison of SUV_max_ and SUV_mean_ as well as the cumulative prednisone dose and current dose of prednisone before PET/CT imaging was performed by linear regression analysis. For table 3, a Mann-Whitney U test was used, adjusting the p values according to Benjamini/Hochberg. In table 4, linear regression analysis was also used for the comparison of visual uptake and quantitative values (SUV_max_).

## Results

### Patients

The study included 60 patients (44 women and 16 men; mean age 66.7±8.9 years). Twenty patients (33.3 %) had a temporal arteritis. Two patients underwent a temporal artery biopsy (TAB), which showed a positive result defined as histopathological infiltration of the vessel wall with inflammatory cells. Forty-four participants were GC naïve and 16 patients received GC at a median of 3.5 days (range: 2–7 days) before ^18^F-FDG PET/CT was performed (prednisone median dose 60 mg, mean cumulative prednisone dose 465.1±538.5 mg, range 30–90 mg per day, one patient with methylprednisolone pulse therapy 1000 mg per day) (table 1).

**Table 1:** Baseline characteristics and clinical patterns

Laboratory findings	C reactive protein mg/L		82.8±63.5 mg/L
	Erythrocyte sedimentation rate 1st hour		65±29 mm/hour
CDS findings	Temporal arteritis		33, 3% (N=20)
Clinical patterns	Cranial symptoms	Headache	40% (N=24)
		Vision impairment	13.3% (N=8)
		Jaw claudication	6.7% (N=4)
		Amaurosis fugax	1.7% (N=1)
	PMR symptoms		31.7% (N=19)
	Systemic symptoms	Weight loss	46.7% (N=28)
		Fatigue	43.3% (N=26)
		Night sweat	28.3% (N=17)
		Cough	25% (N=15)
		Fever	11.7% (N=7)
Treatment	Mean cumulative prednisone dose in GC-treated patients (N=16)		465.1±538.5 mg

CDS, colour Doppler sonography; GC, glucocorticoid; PMR, polymyalgia rheumatica.

At the time of diagnosis, patients presented with a mean CRP of 82.8±63.5 mg/L and ESR (first hour) of 65±29 mm/hour. The major clinical patterns of GCA were weight loss (46.7%), headache (40%), PMR (31.7%), night sweat (28.3%) and cough (25%). Only one patient (GC-treated group) showed no uptake on PET/CT or a pathological CDS examination; the GCA diagnosis was performed by a positive TAB.

### ^18^F-FDG PET/CT findings

#### Vessel involvement

The most commonly affected vessel segments were the central arteries including ascending aorta (72% of patients), followed by the brachiocephalic trunk (62% of patients) as well as the aortic arch and descending aorta in 60% of patients, respectively. The peripheral arteries (left and right subclavian arteries) were involved in 60% and 58% of patients, respectively. The left axillary artery and both carotid arteries were each affected in 57% of patients. Fifty-five per cent of patients showed an involvement of the right axillary artery. The common iliac and femoral arteries were rarely affected (right and left iliac arteries 35% each; right and left femoral arteries 25% each) (table 2).

**Table 2:** Involvement of different arteries

Arteries	GC-naïve patients N=44	GC-treated patients N=16	Total study cohort N=60
Ascending aorta	86% (N=38)	31% (N=5)	72% (N=43)
Aortic arch	73% (N=32)	25% (N=4)	60% (N=36)
Descending aorta	77% (N=34)	13% (N=2)	60% (N=36)
Abdominal aorta	66% (N=29)	19% (N=3)	53% (N=32)
Brachiocephalic trunk	75% (N=33)	25% (N=4)	62% (N=37)
Right subclavian artery	66% (N=29)	38% (N=6)	58% (N=35)
Left subclavian artery	70% (N=31)	31% (N=5)	60% (N=36)
Right axillary artery	64% (N=28)	31% (N=5)	55% (N=33)
Left axillary artery	66% (N=29)	31% (N=5)	57% (N=34)
Right carotid artery	66% (N=29)	31% (N=5)	57% (N=34)
Left carotid artery	68% (N=30)	25% (N=4)	57% (N=34)
Right common iliac artery	48% (N=21)	0% (N=0)	35% (N=21)
Left common iliac artery	45% (N=20)	6% (N=1)	35% (N=21)
Right femoral artery	34% (N=15)	0% (N=0)	25% (N=15)
Left femoral artery	34% (N=15)	0% (N=0)	25% (N=15)

GC, glucocorticoid.

#### Aortic versus peripheral artery involvement in GCA

**Figure 3:**
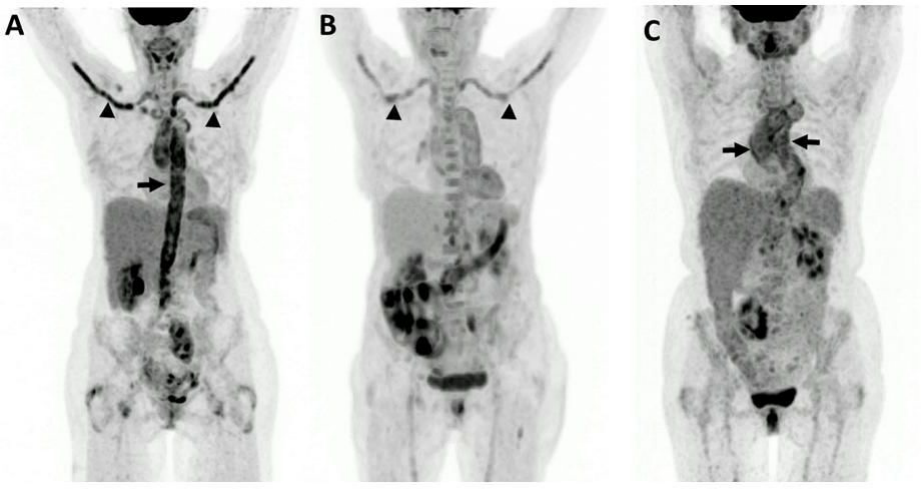
(A) GCA involvement of the aorta (arrow) and its branches (arrowheads), (B) GCA of the supra-aortic arteries (arrows) and (C) GCA of the aortic arch and the thoracic aorta. GCA, giant cell arteritis.

The central and peripheral arteries (aorta and its branches) showed an involvement in 83.3% of patients (50 of 60 patients). A total of 16.7% of patients (10 of 60 patients) had no pathological ^18^F-FDG uptake of the vessels mentioned. An isolated affection of the central arteries (aorta and the brachiocephalic trunk) without any peripheral vessel affection was demonstrated in 20.0% of patients (12 of 60 patients). In one case (1.6%, 1 of 60 patients), only the peripheral arteries were involved (figure 1 and figure 3).

#### Comparison of arterial involvement in GC-naïve and GC-treated patients

GC-naïve patients (95.5%, 42 of 44 patients) had more frequently affected arteries compared with GC-treated participants (50%, 8 of 16 patients). Regarding the involvement of central arteries, the GC-naïve patients revealed a manifestation in 22.7% (10 of 44 patients) compared with the GC-treated patients with 12.5% (2 of 16 patients). The GC-naïve group revealed no isolated involvement of peripheral arteries without a manifestation of central arteries. For the GC-treated group, one patient (6.2%, 1 of 16 patients) was detected with a singular peripheral artery involvement (without manifestation at the central arteries) (figure 1).

GC-naïve patients showed a higher ^18^F-FDG uptake in the central arteries (ascending and descending aorta as well as in the aortic arch, the brachiocephalic trunk) compared with GC-treated patients. A similar result was observed for the peripheral arteries (left subclavian artery, both carotid arteries, left carotid artery and right common iliac artery). The right subclavian artery, both axillary arteries, the left common iliac artery as well as both femoral arteries showed negative mean SUV_max_ (table 3).

**Table 3:** SUV_max_ (maximum standardised uptake values) of the different arteries

Arteries	GC-naïve patients N=44 SUV_max_ mean±SD	GC-treated patients N=16 SUV_max_ mean±SD	P values GC naïve vs GC treated
Ascending aorta	+0.171±0.394	−0.215±0.299	<0.05
Aortic arch	+0.175±0.491	−0.159±0.598	<0.05
Descending aorta	+0.266±0.541	−0.122±0.291	<0.05
Abdominal aorta	+0.405±0.519	+0.017±0.641	<0.05
Brachiocephalic trunk	+0.064±0.524	−0.319±0.392	<0.05
Right subclavian artery	−0.087±0.692	−0.475±0.426	n.s.
Left subclavian artery	+0.041±0.706	−0.388±0.413	n.s.
Right axillary artery	−0.182±0.803	−0.619±0.740	n.s.
Left axillary artery	−0.164±0.835	−0.775±0.718	n.s.
Right carotid artery	+0.041±0.548	−0.213±0.507	n.s.
Left carotid artery	+0.048±0.535	−0.246±0.525	n.s.
Right common iliac artery	+0.032±0.493	−0.269±0.341	n.s.
Left common iliac artery	−0.022±0.554	−0.292±0.702	<0.05
Right femoral artery	−0.043±0.594	−0.229±0.438	n.s.
Left femoral artery	−0.049±0.584	−0.270±0.392	n.s.

Adjusted SUV_max_, which are in relation to the liver and subsequently logarithmised to base 2. Positive values are associated with inflammation in the vessel walls, negative values indicate a lack of inflammation in the vessel walls.

GC, glucocorticoid; n.s., not significant.

#### Comparison of SUV and GC treatment

A negative coefficient of correlation (r=−0.310; p=0.243) was observed between the SUV_max_ and the mean cumulative prednisone dose. The coefficient of correlation between SUV_max_ current dose of prednisone before PET/CT imaging was r=−0.048 (p=0.858) (figure 2).

#### Comparison of clinical patterns and vessel involvement in GCA

**Figure 4:**
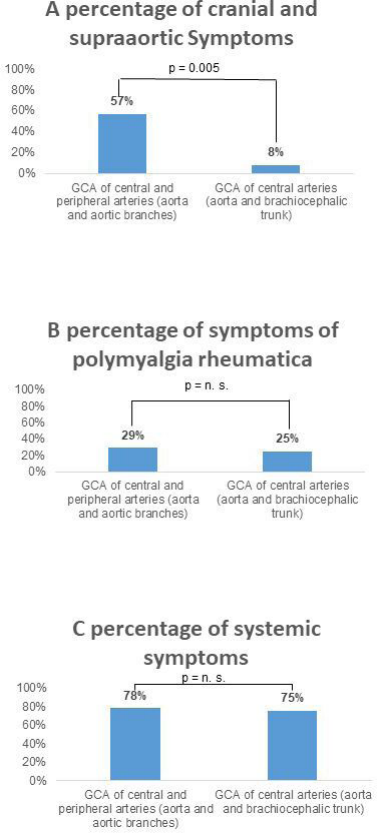
Differentiation of clinical symptoms regarding the affected arterial regions: (A) cranial symptoms (headache, jaw claudication, loss of vision, amaurosis fugax), (B) PMR and (C) systemic symptoms (cough, fever, night sweats, weight loss, fatigue). GCA, giant cell arteritis; n.s., not significant; PMR, polymyalgia rheumatica.

Supra-aortic symptoms (headache, jaw claudication, loss of vision, amaurosis fugax) were more frequent in patients with involvement of the central and peripheral arteries (57 %), than in patients with central arterial involvement (8 %) with a p value of 0.005. In contrast, both groups of vessel involvement showed equal frequency in patients with PMR (central and peripheral arteries 29%; central arteries 25%; p=1) or systemic symptoms (central and peripheral arteries 78%; central arteries 75%; p=1) (figure 4).

#### Comparison of visual uptake values and quantitative SUV

For the total study cohort, the coefficient of correlation between visual uptake and quantitative values (SUV_max_) ranged between r=0.582 (p<0.001) for the left common iliac artery and r=0.780 (p<0.001) for the right axillary artery. Regarding GC-naïve patients, the coefficient of correlation was observed between r=0.504 (aortic arch) and r=0.811 (left femoral artery). A similar result was revealed for the GC-treated patients (brachiocephalic trunk: r=0.327 and left common iliac artery: r=0.909) (table 4).

**Table 4:** Linear regression analysis for the comparison of visual uptake and quantitative values (SUV_max_)

Arteries	Coefficient of correlation between (p values) visual uptake and quantitative values (SUV_max_) total study cohort	Coefficient of correlation between (p values) visual uptake and quantitative values (SUV_max_) GC-naïve patients	Coefficient of correlation between p values) visual uptake and quantitative values (SUV_max_) GC-treated patients
Ascending aorta	0.652 (<0.001)	0.545 (<0.001)	0.655 (0.006)
Aortic arch	0.593 (*<*0.001)	0.504 (<0.001)	0.652 (0.006)
Descending aorta	0.596 (<0.001)	0.543 (<0.001)	0.420 (0.105)
Abdominal aorta	0.675 (<0.001)	0.630 (<0.001)	0.695 (0.003)
Brachiocephalic trunk	0.671 (<0.001)	0.704 (<0.001)	0.327 (0.217)
Right subclavian artery	0.719 (<0.001)	0.701 (<0.001)	0.769 (<0.001)
Left subclavian artery	0.718 (<0.001)	0.699 (<0.001)	0.731 (<0.001)
Right axillary artery	0.780 (<0.001)	0.761 (<0.001)	0.777 (<0.001)
Left axillary artery	0.746 (<0.001)	0.730 (<0.001)	0.683 (0.004)
Right carotid artery	0.650 (<0.001)	0.664 (<0.001)	0.526 (0.036)
Left carotid artery	0.665 (<0.001)	0.640 (<0.001)	0.633 (0.008)
Right common iliac artery	0.612 (<0.001)	0.611 (<0.001)	0.706 (<0.001)
Left common iliac artery	0.582 (<0.001)	0.526 (<0.001)	0.909 (<0.001)
Right femoral artery	0.720 (<0.001)	0.786 (<0.001)	0.720 (<0.001)
Left femoral artery	0.758 (<0.001)	0.811 (<0.001)	0.758 (<0.001)

GC, glucocorticoid; SUV_max_, maximum standardised uptake values.

## Discussion

In recent years, there have been a growing interest and knowledge on GCA with involvement of extracranial territories. As shown in the literature, patients with extracranial GCA are often younger, show less frequently persistent visual disturbances, but are more frequently affected by PMR.^14–16^

Especially in cases with isolated extracranial GCA without any typical cranial manifestations, the diagnosis can be very challenging since symptoms may be non-specific (fever, fatigue, weight loss), resulting in a *delay* of diagnosis and treatment with potential serious consequences.^17^ In addition, involvement of the aorta is associated with a higher risk of aortic dilation later in the course of the disease.^18^ Compared with the general population, patients with aortic aneurysm/dissection have an excess mortality attributed to cardiovascular and pulmonary causes.^19^ According to the data from the French Mortality Registry, GCA-associated deaths are mainly due to aortic aneurysm and dissection, arterial hypertension, diabetes mellitus, infections and coronary artery disease.^20^

Duplex ultrasound is the first-line diagnostic test for patients with suspected GCA. However, due to the anatomical location below bone and air, the technique is of limited value for assessment of the thoracic aorta,^4 21^ which has been the most commonly affected artery in our study. Moreover, extrapolation of GCA manifestations in ultrasound-accessible vessels without their direct detection is problematic. As we have shown in our study, aortic involvement is the most common feature in GCA and some of the patients have isolated aortic involvement, which cannot be detected by an ultrasound only-based diagnostic algorithm. Therefore, it can be problematic to assess only the supra-aortic vessels accessible by ultrasound without evaluating the aorta by imaging, thus possibly overlooking GCA.

Newer imaging techniques such as ^18^F-FDG PET/CT can be of valuable help to identify GCA, in particular in those patients with predominant extracranial manifestations. When we gained clinical experience with GCA in 2008 and 2009, we frequently experienced false-negative MRI results. However, GCA could be well visualised with ^18^F-FDG PET/CT. Since we have a close cooperation with nuclear medicine at our hospital and thus have easy access to PET/CT, we routinely perform ^18^F-FDG PET/CT when GCA is clinically suspected.

As shown by others, ^18^F-FDG PET/CT revealed a high sensitivity (81.8%) and specificity (91.0%) for the assessment of extracranial GCA.^22^ Therefore, based on the available evidence, ^18^F-FDG PET/CT imaging has a high diagnostic value for the detection of LVV and PMR.^8^

Our study showed a predominant involvement of the central arteries (ascending aorta, the aortic arch, descending aorta, brachiocephalic trunk) and the supra-aortic branches. This is in accordance with a study by Soriano *et al*, in which the highest SUV_max_ in ^18^F-FDG PET/CT scans were found at the ascending aorta and the aortic arch, followed by the descending and abdominal aorta.^23^ Also, Imfeld *et al* reported that the best discrimination between patients with GCA and controls was achieved for PET/CT findings within the supra-aortic arteries.^24^ Furthermore, Imfeld *et al* showed recently that ^18^F-FDG PET/CT-assessed vasculitis was mainly present in the supra-aortic vessels and the aorta with vertebral and common carotid arteries being the most commonly affected vessel segments.^25^ De Boysson *et al* compared patients with large-vessel involvement diagnosed with imaging with patients without large-vessel involvement.^18^ The retrospective multicentre study recognised that patients with large-vessel involvement were younger (p<0.0001), more likely to be women (p=0.01), and showed fewer cephalic symptoms (p<0.0001) and PMR (p=0.001) but more extracranial vascular symptoms (p=0.05) than patients without large-vessel involvement. The authors concluded that large-vessel involvement is a distinct epidemiological, clinical and prognostic spectrum of GCA with a higher risk of aortic dilation, especially in previously inflamed vascular segments.

In different studies, the detection rate of PET/CT or PET for LVV (GCA and Takayasu arteritis) has been described between 69% and 92%, although in some cases patients were treated with GC and this is potentially associated with a lower detection rate in PET/CT or PET.^26–28^ These results are in accordance with our findings (positive PET/CT in 83.3% of patients). It should be noted that recent studies included GC-naïve as well as GC-treated patients. In the GC-naïve group, 95.5% of patients presented a positive PET/CT finding (GC-treated group: 50.0%). Our study also revealed that the use of GC prior to ^18^F-FDG PET/CT is associated with a marked decrease in the inflamed vascular segments and thus reducing its diagnostic power. Imfeld *et al* showed a significantly impacted performance of ^18^F-FDG PET/CT as early as 3 days after treatment initiation in the abdominal aorta, and 10 days in the other examined arteries.^24^ This is also exactly in line with the findings of Nielsen *et al* reporting that within 3 days of high-dose GC treatment, ^18^F-FDG PET/CT can diagnose GCA with high sensitivity.^29^ After 10 days of treatment, FDG PET/CT sensitivity decreased significantly.

Furthermore, our study revealed a negative correlation between SUV_max_ and mean cumulative GC dose, whereby a correlation between SUV_max_ and current dose of GC before PET/CT imaging was found. These data support the fact that the cumulative GC dose is associated with a reduced SUV uptake in PET/CT. It should be noted that the single GC dose before PET/CT did not lead to a decreased SUV uptake.

Steroid treatment also affects the presence of the halo sign, the most diagnostic feature of cranial GCA on Doppler ultrasound, which may disappear within a few days to a few months in 95% of cases. Therefore, the sensitivity of Doppler ultrasound may be as low as 50% 2 days after the start of GC.^30^ These results underline the significant influence of high-dose GC therapy on ^18^F-FDG PET/CT findings in GCA and the importance of its timely use in order to avoid debilitating GCA complications with a limited effect on diagnostic accuracy.

We used the qualitative visual scoring method comparing the vascular ^18^F-FDG uptake with the liver uptake (vascular to liver uptake ratio) as recommended by Stellingwerff *et al*.^13^ The latter showed a sensitivity of 92% and a specificity of 91% by comparing vessel uptake versus liver uptake. The evaluation of the vessels judged on first impression without a comparison revealed a sensitivity and specificity of 75% and 98% in the detection of GCA in PET/CT.^13^ Furthermore, the liver is the common reference in the PET vascular activity score which compares the vessel uptake in relation to the liver uptake.^31^ In summary, the diagnostic performance of PET/CT analysis can be increased by comparing the vessel uptake with the liver uptake. Additionally, the diagnostic performance of PET/CT can be stabilised by using the SUV_max_ ratio between the vessels and liver (SUV_vessel-max_/SUV_liver-max_) with a sensitivity and specificity of 92%.^13^ In this context, it should be noted that the comparison of the vessel uptake with the blood uptake revealed an inhomogeneous sensitivity (comparison vessel uptake vs blood uptake inferior vena cava: 92% and comparison vessel uptake vs blood uptake superior vena cava: 75%) as well as specificity (comparison vessel uptake vs blood uptake inferior vena cava: 73% and comparison vessel uptake vs blood uptake superior vena cava: 96%).^13^ PET SUV are usually log-normal distributed.^32^ Since many statistical tests require normal distributed data or residuals, SUV were log-transformed prior to all statistical analyses. This does not contradict approaches that use target/liver ratios, since the ratio normalisation of these approaches also relies on the log-normal distribution of SUV.

In a subanalysis, we performed a comparison between the visual uptake values and the quantitative SUV showing a moderate and high positive coefficient of correlation between both values. Additionally, our study showed lower and negative SUV_max_ in the GC-treated group reflecting a lower GC uptake compared with the liver. This phenomenon can be explained with a significantly higher liver uptake in patients under treatment with GC,^13^ resulting in a reduced SUV uptake as ratio between the vessel and liver. Because of the increased liver uptake under GC therapy and the reduced uptake in the vessel wall under GC therapy, the measurement of SUV as a ratio between vessel wall and liver is not considered useful.

A limitation of our study is the low number of patients. However, GCA is a rare disease with small numbers of patients. Therefore, imaging studies are usually performed in populations with LVV, which includes GCA and Takayasu arteritis,^33^ in order to achieve meaningful patient numbers. Given these two disease patterns, such studies may provide differential results regarding the diagnostic value of PET/CT compared with a GCA-only cohort. The advantage of our study is that the number of patients, although quite small, is very large for a GCA cohort.

Furthermore, arterial vessel walls are relatively thin and can also pulsate (=move) during the PET/CT scan. Therefore, the uptake in the vessel can vary between two or more measurement voxels. Although the SUV_max_ is a comparatively robust parameter, partial volume and blood effects are highly probable, so that the measured values could be underestimated (false negative).

In addition, patients with cranial GCA may potentially be under neurological care, so these patients might be under-represented in a rheumatological patient population.

## Conclusion

Due to the increasing use of modern whole-body imaging techniques like ^18^F-FDG PET/CT, greater awareness of extracranial GCA has been raised. In these patients, systemic symptoms and an acute inflammatory blood profile may be the only presenting features and the classic cranial signs might be absent. In our study, approximately 22% of patients with GCA had an isolated involvement of the aorta which cannot be detected by Doppler ultrasound. Therefore, imaging of the aorta should be performed if GCA is suspected. ^18^F-FDG PET/CT is of special value when there are hints for extracranial GCA because it allows the detection of large-vessel involvement. Since the use of GC is associated with a marked decrease in vascular ^18^F-FDG uptake and therefore lowers the sensitivity and specificity of the technique, ^18^F-FDG PET/CT should be performed as soon as possible.

## Data Availability

Data are available upon reasonable request.
